# Co-existing patterns of MRI lesions were differentially associated with knee pain at rest and on joint loading: a within-person knee-matched case-controls study

**DOI:** 10.1186/s12891-020-03686-4

**Published:** 2020-10-06

**Authors:** Qiang Liu, Nancy E. Lane, David Hunter, Dan Xing, Zhikun Li, Jianhao Lin, Yuqing Zhang

**Affiliations:** 1 Peking University People’s Hospital, Arthritis Clinic and Research Center, No.11 Xizhimen South Road, Xicheng District, Beijing, 100044 China; 2 Division of Rheumatology, Allergy, and Immunology, Massachusetts General Hospital, 55 Fruit St, Boston, MA 02114 USA; 3 Center for Musculoskeletal Health, University of California, Davis School of Medicine, Sacramento, USA; 4 Institute of Bone and Joint Research, The Kolling Institute, Sydney Medical School, The University of Sydney, Sydney, Australia

**Keywords:** MRI, Pattern, Pain, Joint loading, Knee, Osteoarthritis

## Abstract

**Background:**

To assess the association of co-existing MRI lesions with knee pain at rest or on joint loading.

**Methods:**

We included participants from Osteoarthritis Initiative whose pain score, measured by WOMAC sub-scales, differed by ≥1 point at rest (in bed at night, sitting/lying down) or on joint loading (walking, stairs) between two knees. Cartilage morphology, bone marrow lesions, meniscus extrusion, meniscus morphology, Hoffa’s synovitis and synovitis-effusion were assessed using the compartment-specific MRI Osteoarthritis Knee Score. We performed latent class analyses to identify subgroups of co-existing MRI lesions and fitted a conditional logistic regression model to examine their associations with knee pain.

**Results:**

Among 130 eligible participants, we identified five subgroups of knees according to patterns of co-existing MRI lesions: I. minimal lesions; II. mild lesions; III. moderate morphological lesions; IV. moderate multiple reactive lesions; and V. severe lesions. Compared with subgroup I, the odds ratios (ORs) and 95% confidence intervals (CI) of greater pain in bed at night were 1.6 (0.3, 7.2), 2.2 (0.5, 9.5), 6.2 (1.3, 29.6) and 11.2 (2.1, 59.2) for subgroups II-V, respectively. A similar association was observed between aforementioned subgroups and pain with sitting/lying down. The ORs (95% CI) of greater pain with walking were 1.0 (reference), 1.7 (0.5, 6.1), 0.7 (0.2, 2.3), 5.0 (1.4, 18.6) and 7.9 (2.0, 31.5) for subgroup I-V, respectively. The corresponding analysis for pain on stairs showed similar results.

**Conclusions:**

Distinct patterns of co-existing MRI lesions have different implications for the pathogenesis of osteoarthritic knee pain occurring with/without joint loading.

## Background

Pain at rest and pain on joint loading are two different manifestations of knee pain in osteoarthritis [1–3]. Pain at rest has been a marker for more severe cases of knee OA and a criterion utilized for the recommendation of total knee replacement (TKR) [4, 5]. Pain on joint loading, e.g., pain with walking, is among the most commonly reported symptoms experienced by patients with knee OA [6], and consequently contributes to physical disability [7–9]. These two types of pain are differentially associated with other clinical variables and response to treatment [3, 10, 11]. For example, neuropathic pain is more strongly associated with pain at rest than pain on joint loading among people with end-stage hip and knee OA [10]. Additionally, patients with higher knee pain at rest are more likely to have less favorable pain relief after TKR [3], whereas pain on joint loading is improved significantly compared with pain at rest among patients treated with TKR or placebo [3, 11]. Collectively, these findings suggest that pain at rest and pain on joint loading may have different underlying mechanisms and corresponding risk factors. However, to our knowledge, despite the different clinical relevance and impact on patients, differential risk factors for these two types of osteoarthritic knee pain are poorly understood.

Knee OA is a disease of the whole joint featured by structural changes in a number of tissues including cartilage, meniscus, synovium and subchondral bone. These lesions can be identified and assessed in a compartment-specific semi-quantitative way on MRI [12]. Studies on different MRI structural lesions have primarily focused on individual MRI lesions and knee OA outcomes [13]. Focusing on a single MRI feature or structural lesion may be overly simplistic as it does not give a comprehensive picture of the relation between MRI lesions and pain in this whole joint disease. For example, articular cartilage is incapable of directly generating pain because it is aneural. However, cartilage defects, in isolation from other tissues, have been reported to be associated with knee pain in OA [14, 15]. Such an association may limit insights into the pathogenesis of osteoarthritic knee pain.

Studying the relation of multiple MRI lesions to knee pain in OA is methodologically challenging. First, many factors that account for an individual’s pain response, such as central hypersensitivity, are either not collected or not controlled for in most observational studies [16]; thus the validity of study findings related to an individual’s pain could be affected by residual confounding. A within-person knee-matched case-control study design has been proposed to eliminate person-level confounders and improve the validity of study findings [17, 18]. Second, to include all MRI lesions into a multivariable regression model and obtain their “independent” effects may be problematic in the absence of knowledge regarding the temporal sequence among the occurrences of MRI lesions. Some effect estimates represent the total effect, and others direct effects, according to the chronology of the occurrence [19, 20]. An alternative strategy is to identify the patterns of co-existing MRI lesions and examine their relation to knee pain in OA [21], which is attractive because model building with many highly correlated predictors can be otherwise nearly impossible. To date, there are no published studies that have utilized these approaches to examine the associations of multiple MRI lesions with subtypes of osteoarthritic knee pain. Therefore, we conducted a within-person knee-matched case-control study to determine if distinct patterns of co-existing MRI lesions have differential associations with knee pain at rest and on joint loading using data collected from the Osteoarthritis Initiative (OAI).

## Methods

### Study sample

The OAI is a longitudinal cohort study of participants with or at high risk of knee OA. At baseline, the OAI cohort included 4796 subjects aged 45–79 years who were recruited from four sites, Columbus, Ohio, Providence, Rhode Island, Baltimore, Maryland and Pittsburgh, Pennsylvania. Annual assessments included the questionnaires, clinical examination and imaging.

### Knee pain at rest and knee pain on joint loading

We used the knee-specific Western Ontario and McMaster Universities Osteoarthritis Index (WOMAC) pain sub-scales (0–5) to define knee pain at rest and on joint loading. The WOMAC pain sub-scales measure the severity of pain occurring in five scenarios during the past 7 days. For the current study, we used the items of pain with sitting/lying down and pain in bed at night as two separate measures of knee pain at rest, and those of pain with walking and pain on stairs as measures of knee pain on joint loading.

### MRI assessments

MRI readings of structural lesions of knee OA, including cartilage morphology (CartM), bone marrow lesion (BML), synovitis-effusion, Hoffa’s synovitis (HFS), meniscus morphology (MM) and meniscus extrusion (MExt), were performed using the compartment-specific semi-quantitative MRI Osteoarthritis Knee Score (MOAKS) in the OAI [12]. Scores for BML and CartM were applied in subregions for each lesion in both the tibiofemoral joint (TFJ) and patellofemoral joint (PFJ), denoted by TFJ-BML, PFJ-BML, TFJ-CartM and PFJ-CartM, respectively. We only pooled data from the knee MRI scans that were read by Boston Imaging Core Laboratory group. We restricted our analyses to those who had MRI readings for both knees in one visit.

### Statistical analysis

**Fig. 1:**
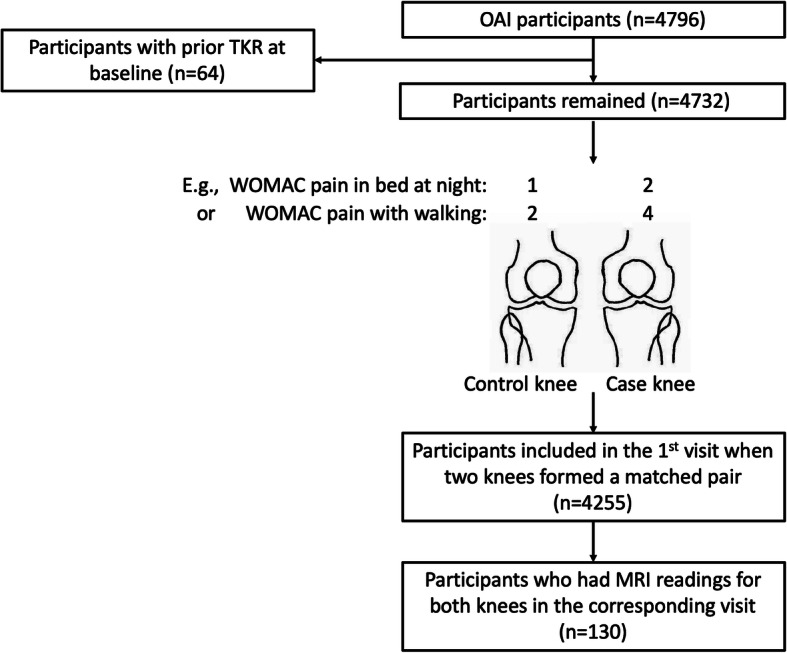
Osteoarthritis Initiative participants included in final analyses until 48-month follow-up. OAI = Osteoarthritis Initiative, TKR = Total knee replacement, WOMAC = the Western Ontario and McMaster Universities Osteoarthritis Index, E.g. = For example

The outcome of interest is a greater score of each of WOMAC pain sub-scales respectively. We identified a matched pair of case and control knees within one participant if his/her two knees differed by ≥1 point in at least one score of knee pain at rest or on joint loading (Fig. 1). Given that the comparison is between two knees within one person, we consider a discordance of ≥1 point as a meaningful difference which is consistent with the magnitude of acute pain flare evoked by an exercise session [22]. Participants were included when their knees formed a matched pair in the earliest visit between baseline and the 48-month visit. Each participant was included only once.

Scores for MRI features were from the same visit when a pair of knees was included. We used the worst CartM score from among 12 subregions in the TFJ (medial and lateral trochlea, medial and lateral central femur, medial and lateral posterior femur; medial anterior tibia, medial central tibia, medial posterior tibia, lateral anterior tibia, lateral central tibia and lateral posterior tibia) and among 2 subregions in the PFJ (medial and lateral patella on the axial view) to represent the severity of cartilage damage in each of the tibiofemoral and patellofemoral joints, respectively. The worst BML score for size from among the aforementioned 12 subregions in the TFJ and among the aforementioned 2 subregions in the PFJ was used to represent the severity of BML in each joint, respectively. The worst MM score from among 6 subregions (medial anterior, body, and posterior; lateral anterior, body, and posterior) and the worst MExt score from among 2 areas (medial and lateral) in 2 views (coronal and sagittal) were used to represent the severity of these lesions in the whole knee.

We performed latent class analysis (LCA) using “gsem” STATA procedure that fits a generalized structural equation model with categorical latent variables to identify subgroups representing distinct patterns of co-existing MRI lesions based on the prevalence and severity of CartM and BML in the TFJ and PFJ, as well as EFF, HFS, MM and MExt in the whole knee. If the proportion of knees with a score level of an MRI lesion was less than 3%, we collapsed those knees to the adjacent lower score level to avoid unstable estimations due to sparse data. We fitted the LCA models with 2–6 subgroups and chose the model according to the following criteria: 1) to have a meaningful clinical relevance; 2) the lowest Akaike information criterion or Schwarz’s Bayesian information criterion to identify the best model fit [23]; 3) to have sufficient numbers of knees (≥10% of the sample) in each subgroup (Table 1). We assigned each knee to the subgroup with the maximum posterior probability generated from LCA model. We then fitted a conditional logistic regression model to deal with the dependence of two knees within one subject in a separate analysis of pain at rest and pain on joint loading. We performed test of homogeneity of odds ratios (ORs) using “tabodds” STATA procedure. Subjects who had a TKR or had missing values for variables of interest in either knee were excluded. All statistical analyses were performed using Stata/SE 15.1 (StataCorp, Texas, USA).

**Table 1:** Goodness-of-fit statistics for latent class models

No. subgroups	AIC	BIC	Average posterior probability
2	4955.023	5044.04	96.1
3	4827.233	4948.296	95.2
4	4778.447	4931.556	95.3
5	485.4094	542.3803	96.0
6	953.0936	1010.065	96.6

*AIC* Akaike information criterion, *BIC* Schwarz’s Bayesian information criterion

## Results

Among 130 eligible participants whose MRI-lesion assessments were available, 60% were women, their mean age was 63.8 years, more than 80% of participants were White, and the mean BMI was 29.6 kg/m^2^. Among 260 knees from 130 eligible participants, the prevalence of Kellgren/Lawrence grade 0, 1, 2, 3 and 4 was 15.8, 28.0, 19.7, 24.8 and 11.8%, respectively.

### Patterns of co-existing MRI lesions

**Fig. 2:**
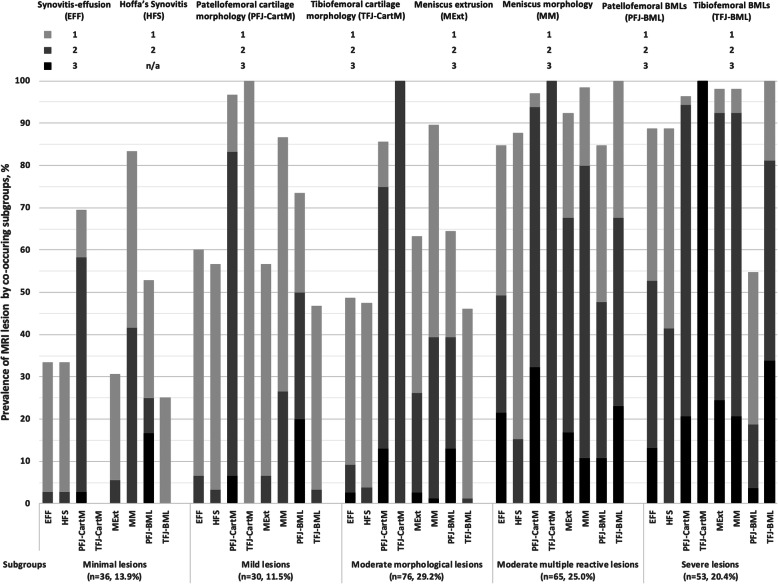
Estimated prevalence and severity of MRI features by subgroups of co-existing MRI lesions. Values of 1, 2 and 3 as depicted by colored squares and bars in figure represent the severity metrics of MRI lesions

We identified five subgroups of knees based on the patterns of co-existing MRI lesions (Table 1): I (*n* = 36, 13.9%), II (*n* = 30, 11.5%), III (*n* = 76, 29.2%), IV (*n* = 65, 25.0%) and V (*n* = 53, 20.4%) (Fig. 2). The average posterior probability of membership was 1.00 for subgroups I, II and V, 0.93 for subgroup III and 0.92 for subgroup IV, respectively, suggesting that subgroup assignment was acceptably unambiguous.

The patterns of co-existing MRI lesions are depicted in Table 2 and Fig. 2. In knees of subgroup I, there was no TFJ cartilage damage, whereas all knees of subgroup II had mild TFJ-CartM. All knees of subgroup III and IV had moderate TFJ-CartM, while the prevalence of severe TFJ-CartM was 100% in subgroup V. The prevalence and severity of MM and MExt also increased from subgroup I to subgroup V. Compared with subgroup III, subgroup IV was featured by a higher prevalence and greater severity of EFF, HFS and TFJ-BML. EFF, HFS and TFJ-BML often coexisted and their prevalence and severity were highly correlated. The prevalence and severity of PFJ-CartM and PFJ-BML was not correlated with that of TFJ-CartM. For example, although knees of subgroup V had greater TFJ-CartM than knees of subgroup IV, the prevalence and severity of PFJ-CartM and PFJ-BML was lower in subgroup V. Based on the differences in prevalence and severity of MRI lesions across subgroups, we labeled them as I: minimal lesions, II: mild lesions, III: moderate morphological lesions, IV: moderate multiple reactive lesions and V: severe lesions.

**Table 2:** Prevalence of MRI lesions according to latent subgroups of knees^a^

MRI features	Score	Subgroup I: Minimal lesions	Subgroup II: Mild lesions	Subgroup III: Moderate morphological lesions	Subgroup IV: Moderate multiple reactive lesions	Subgroup V: Severe lesions
Synovitis-Effusion	0	66.7	40.0	51.3	15.4	11.3
	1	30.6	53.3	39.5	35.4	35.9
	2	2.8	6.7	6.6	27.7	39.6
	3	0.0	0.0	2.6	21.5	13.2
Hoffa’s Synovitis	0	66.7	43.3	52.6	12.3	11.3
	1	30.6	53.3	43.4	72.3	47.2
	2	2.8	3.3	4.0	15.4	41.5
Patellofemoral cartilage morphology	0	30.6	3.3	14.5	3.1	3.8
	1	11.1	13.3	10.5	3.1	1.9
	2	55.6	76.7	61.8	61.5	73.6
	3	2.8	6.7	13.2	32.3	20.8
Tibiofemoral cartilage morphology	0	100.0	0.0	0.0	0.0	0.0
	1	0.0	100.0	0.0	0.0	0.0
	2	0.0	0.0	100.0	100.0	0.0
	3	0.0	0.0	0.0	0.0	100.0
Meniscus extrusion	0	69.4	43.3	36.8	7.7	1.9
	1	25.0	50.0	36.8	24.6	5.7
	2	5.6	6.7	23.7	50.8	67.9
	3	0.0	0.0	2.6	16.9	24.5
Meniscus morphology	0	16.7	13.3	10.5	1.5	1.9
	1	41.7	60.0	50.0	18.5	5.7
	2	41.7	26.7	38.2	69.2	71.7
	3	0.0	0.0	1.3	10.8	20.8
Patellofemoral bone marrow lesions	0	47.2	26.7	35.5	15.4	45.3
	1	27.8	23.3	25.0	36.9	35.9
	2	8.3	30.0	26.3	36.9	15.1
	3	16.7	20.0	13.2	10.8	3.8
Tibiofemoral bone marrow lesion	0	75.0	53.3	54.0	0.0	0.0
	1	25.0	43.3	44.7	32.3	18.9
	2	0.0	3.3	1.3	44.6	47.2
	3	0.0	0.0	0.0	23.1	34.0

^a^Values are displayed in percentage

### Subgroups of co-existing MRI lesions and knee pain at rest

The risk of having greater knee pain at rest increased from subgroup II to subgroup V compared with subgroup I (Table 3). The ORs and 95% confidence intervals (CI) of greater pain in bed at night were 1.0 (reference), 1.6 (0.3, 7.2), 2.2 (0.5, 9.5), 6.2 (1.3, 29.6) and 11.2 (2.1, 59.2) from subgroup I to subgroup V, respectively (P for test of homogeneity =0.056), indicating that risk of pain at rest varied among the different subgroups. Similar results were observed when knee pain at rest was measured using WOMAC subscale of pain that occurs when subjects were sitting/lying down. The corresponding ORs were 1.0 (reference), 1.0 (0.2, 5.6), 3.1 (0.7, 14.4), 14.4 (2.5, 81.7) and 28.9 (4.5, 184.0), respectively (*P* for test of homogeneity < 0.001) (Table 3).

**Table 3:** Subgroups of co-existing MRI lesions and knee pain at rest

	Knee pain at rest					
	Pain in bed at night			Pain with sitting/lying down		
Subgroups	Control knee	Case knee	OR (95% CI)	Control knee	Case knee	OR (95% CI)
I. Minimal lesions	13	7	1.0 (Reference)	16	8	1.0 (Reference)
II. Mild lesions	14	8	1.6 (0.3, 7.3)	14	4	1.1 (0.2, 5.6)
III. Moderate morphological lesions	29	22	2.2 (0.5, 9.5)	31	20	3.1 (0.7, 14.4)
IV. Moderate multiple reactive lesions	22	28	6.2 (1.3, 29.6)	20	31	14.4 (2.5, 81.7)
V. Severe lesions	14	27	11.2 (2.1, 59.2)	13	31	28.9 (4.5, 184.0)
	*P* for test of homogeneity = 0.056			*P* for test of homogeneity < 0.001		

*OR* Odds ratio, *CI* Confidence interval

Figures are numbers unless stated otherwise

### Subgroups of co-existing MRI lesions and knee pain on joint loading

Knees of subgroup IV and V had a higher risk of greater knee pain with walking compared with subgroup I (Table 4). The ORs of greater pain with walking were 1.0 (reference), 1.7 (0.5, 6.1), 0.7 (0.2, 2.3), 5.0 (1.4, 18.6) and 7.8 (2.0, 31.5) from subgroup I to subgroup V, respectively (P for test of homogeneity =0.001), suggesting that risk of pain with walk varied statistically significantly among five subgroups. Using WOMAC item for pain on stairs as one other measure of knee pain on joint loading, the corresponding ORs were 1.0 (reference), 0.9 (0.3, 3.3), 0.8 (0.3, 2.5), 2.4 (0.7, 7.4) and 6.6 (1.8, 23.9), respectively (*P* for test of homogeneity =0.003) (Table 4).

**Table 4:** Subgroups of co-existing MRI lesions and knee pain on joint loading

	Knee pain on joint loading					
	Pain with walking			Pain on stairs		
Subgroups	Control knee	Case knee	OR (95% CI)	Control knee	Case knee	OR (95% CI)
I. Minimal lesions	16	11	1.0 (Reference)	19	15	1.0 (Reference)
II. Mild lesions	15	14	1.7 (0.5, 6.1)	16	10	0.9 (0.3, 3.3)
III. Moderate morphological lesions	45	21	0.7 (0.2, 2.3)	44	27	0.8 (0.3, 2.5)
IV. Moderate multiple reactive lesions	24	37	5.0 (1.4, 18.6)	30	34	2.4 (0.7, 7.4)
V. Severe lesions	16	33	7.8 (2.0, 31.5)	15	38	6.6 (1.8, 23.9)
	*P* for test of homogeneity = 0.001			*P* for test of homogeneity = 0.003		

*OR* Odds ratio, *CI* Confidence interval

Figures are numbers unless stated otherwise

## Discussion

Among the OAI participants with or at high risk of knee OA, we identified five distinct patterns of co-existing MRI lesions that were differentially associated with subtypes of knee pain in OA. These findings not only allow a glimpse of distinct clinically relevant pathways leading to knee pain, but also provide insights into the pathogenesis of knee pain in OA.

When we compared subgroup III (moderate morphological lesions) with subgroup I (minimal lesions), the results suggested that morphologic lesions of cartilage and meniscus were not significantly associated with knee pain at rest or knee pain on joint loading. Aneural hyaline articular cartilage and the meniscus do not generate pain directly, or appear to have a limited role in the pathogenesis of pain. Although there is data that vascular penetration and nerve growth after a meniscal tear may be a source of pain [24], osteoarthritic knees with a meniscal tear are not more painful than those without a tear [25]. Therefore, meniscal tears are an unlikely immediate source of knee pain in OA [26], underlying the large body of evidence that arthroscopic surgery targetting meniscus was no better than conservative interventions such as exercise therapy in pain relief [27].

BMLs reflect increased focal loading in the subchondral bone [28]. Synovitis-effusion is thought to be an inflammatory reaction that could be triggered by structural damage. Consistent with the published study [21], we found that synovitis-effusion and TFJ-BML often coexisted, especially with TFJ-CartM. Meanwhile, the severity of these lesions correlated well in subgroups. In the current study, the difference between subgroup III (moderate morphological lesions) and subgroup IV (moderate multiple reactive lesions) that have the same level of TFJ-CartM mostly lies in the prevalence and severity of EFF, HFS and TFJ-BML. Knees of subgroup IV had a higher risk of greater knee pain at rest and on joint loading than knees of subgroup III, supporting other studies that have reported that synovitis-effusion and TFJ-BML may be the major sources of knee pain in OA [18, 29]. Given that pain is the key reason for seeking medical care [30], these lesions should be considered as clinically relevant markers of symtomatic knee OA. A recent study showed that improvement of synovitis on MRI following a transcatheter arterial embolization was associated with a significant reduction in WOMAC pain among patients with mild to moderate radiographic knee OA [31]. Studies have also shown that BMLs can be reduced by zoledronic acid or prostacyclin analogue iloprost [32, 33]. Our findings highlight the importance of modifying either synovitis-effusion or BMLs to reduce knee pain and consequent pain-related disability.

The role of PFJ in the surgical treatment of knee OA remains controversial [34–37]. For example, there is evidence that preoperative PFJ OA or anterior knee pain does not compromise the outcome or survival of a medial unicompartmental knee replacement [34, 35]. Additionally, at the time of a TKR, patellar retention was not statistically significantly associated with the risk of incident postoperative anterior knee pain, compared with patellar resurfacing for patients receiving TKR [36]. Based on latent class analysis, we identified the co-existing pattern of BMLs and CartM that are located separately in PFJ and TFJ, allowing us to examine their differential contributions to osteoarthritic knee pain. We found that knees with greater TFJ-CartM could have lower prevalence and severity of PFJ-CartM and PFJ-BML (e.g., subgroup V versus subgroup IV, or subgroup III versus subgroup II). In contrast, subgroup V (severe lesions) had larger ORs of both knee pain at rest and on joint loading than subgroup IV (moderate multiple reactive lesions), suggesting that lesions in PFJ were not significantly associated with subtypes of knee pain (e.g., knee pain on stairs). Our findings, if confirmed, are informative for the management of patella during surgical treatment from the perspective of structural pathogenic source of pain.

In contrast to knee pain at rest that represents pain occurring without mechanical stimuli, knee pain on joint loading is a symptom in response to localized stress. Interestingly, joint loading can act as either a source of pain or a remedy for pain among individuals with knee OA [38]. Animal studies showed that joint loading may attenuate structural damage in OA, depending on the amount and frequency [39, 40]. Meanwhile, there is evidence from human studies indicates that exercise, such as land-based training, is effective in the management knee OA [41, 42]. In the present study, we observed larger and more skewed ORs for pain at rest than for pain on joint loading. When we compared the subgroup III (moderate morphological lesions) with the subgroup I (minimal lesions), the ORs of knee pain on joint loading were less than 1.0 (i.e., 0.7 for pain with walking and 0.8 for pain on stairs) whereas the ORs of knee pain at rest were over 2.0 (i.e., 2.2 for pain in bed at night and 3.1 for pain with sitting/lying down). Collectively, these findings may add additional evidence that mechanical loading may attenuate knee pain for patients with symptomatic knee OA. It should be noted that there are other explanations. For example, various methods of distraction potentially including physical activity can be used as a modifying tactic to reduce pain [43].

Our study has several strengths. We assessed knee pain at rest and knee pain on joint loading using four items of WOMAC pain sub-scale, allowing us to separately look at knee pain in response to different pathogenic sources. Since our study design allowed the assessment of the pain difference in two knees within the same person over a short period of time, we believe that participants should be able to tell a one-point difference. Moreover, in the absence of knowledge of the temporal sequence of MRI lesions, we fitted latent class analysis to identify subgroups representing distinct patterns of co-existing MRI lesions. Although our study is subjective to limitations of a case-control study such a selection bias, as an advance over previous studies [44, 45], we were able to examine the differential association of multiple MRI lesions localized in different compartments with knee pain occurring with or without joint loading. Finally, person-level confounders were eliminated using a within-person knee-matched case-control study design; thus, the validity of the study findings was improved.

Limitations of the present study should be noted. First, although we identified patterns of co-existing MRI lesions, we still cannot tell the temporal sequence of occurrence of these MRI lesions. For example, TFJ-BML often coexisted with synovitis-effusion in subgroups [21], making it difficult to sort out their differential contributions to knee pain. Second, uncertainty in subgroup membership assignment might lead to potentially biased effect estimate, albeit the maximum-probability approach. However, the lowest average posterior probability of membership was above 0.92 among all subgroups, suggesting that subgroup assignment was robust. Third, each WOMAC pain subscale item (0–5) that we used to identify case and control knees has not been validated separately from the pain subscale as a whole. Forth, we used the difference of ≥1 point for both pain at rest and pain on joint loading regardless of potentially different variance in the score for each item [46]. Finally, because we adopted a within-person case-control study design, we were not able to examine the association of person-level factors, such as central hypersensitivity and coping strategy, with knee pain in OA.

## Conclusions

In conclusion, co-existing patterns of MRI lesions were identified and differentially associated with knee pain at rest and on joint loading in OA. Morphological lesions of cartilage and meniscus were not statistically significantly associated with pain. Synovitis-effusion and TJF-BML were highly correlated and appeared to be the major sources of pain. PFJ lesions had a limited role in the pathogenesis of osteoarthritic knee pain. These findings are informative for optimizing a treatment strategy targeting the pathogenic sources of osteoarthritic knee pain.

## Data Availability

The datasets generated and/or analysed during the current study are available in the OAI repository, https://nda.nih.gov/oai.

## Abbreviations

OA: Osteoarthritis
TKR: Total knee replacement
OAI: The Osteoarthritis Initiative
WOMAC: Western Ontario and McMaster Universities Osteoarthritis Index
AIC: Akaike information criterion
BIC: Schwarz’s Bayesian information criterion
CartM: Cartilage morphology
BML: Bone marrow lesion
EFF: Synovitis-effusion
HFS: Hoffa’s synovitis
MM: Meniscus morphology
MExt: Meniscus extrusion
MOAKS: MRI Osteoarthritis Knee Score
TFJ: Tibiofemoral joint
PFJ: Patellofemoral joint
LCA: Latent class analysis
ORs: Odds ratios
CI: Confidence interval
